# Neonatal α-Ketoglutaric Acid Gavage May Potentially Alleviate Acute Heat Stress by Modulating Hepatic Heat Shock Protein 90 and Improving Blood Antioxidant Status of Broilers

**DOI:** 10.3390/ani14152243

**Published:** 2024-08-01

**Authors:** Vaishali Gupta, Akshat Goel, Chris Major Ncho, Chae-Mi Jeong, Yang-Ho Choi

**Affiliations:** 1 Division of Animal Science, Gyeongsang National University, Jinju 52828, Republic of Korea; vaishali2020@gnu.ac.kr (V.G.); genesakshat@gnu.ac.kr (A.G.); major159@gnu.ac.kr (C.M.N.); wjdcoa@gnu.ac.kr (C.-M.J.); 2 Division of Applied Life Sciences (BK21 Four Program), Gyeongsang National University, Jinju 52828, Republic of Korea; 3 Institute of Agriculture and Life Sciences, Gyeongsang National University, Jinju 52828, Republic of Korea

**Keywords:** acute heat stress, broilers, alpha-ketoglutaric acid, gavage feeding, hepatic gene expression

## Abstract

**Simple Summary:**

Heat stress (HS) poses a significant challenge to the poultry industry and leads to various behavioral, physiological, metabolic, and immunological changes. HS is a progenitor of oxidative stress, causing cellular damage and metabolic changes. The current study found that gavage feeding of α-ketoglutaric acid (AKG) might help mitigate plasma oxidative stress and modulate hepatic HSP expression in broilers under acute heat stress (AHS).

**Abstract:**

This study investigated the effect of neonatal α-ketoglutaric acid (AKG) gavage feeding on broilers. The first experiment was conducted to determine the effect of AKG on day-old broilers. A total of seventy-two-day-old Ross 308 broiler chicks were divided into four treatment groups: (i) Two groups of chicks with gavage feeding of 0.6 mL of distilled water (DDW) for four consecutive days (CON); (ii) chicks fed with 0.6 mL of 0.1% AKG dissolved in DDW on the day of hatch (AL) followed by 0.2%, 0.3%, and 0.4% for three consecutive days; and (iii) chicks fed with 0.6 mL of 0.2% AKG dissolved in DDW on the day of hatch (AH) followed by 0.4%, 0.6%, and 0.8% for three consecutive days. Twenty-four hours after the first gavage feeding, six birds per treatment were slaughtered to study the organ development. Chicks fed with AKG showed higher absolute (*p* = 0.015) and relative (*p* = 0.037) weights of the gizzard. The AH group had higher absolute (*p* = 0.012) and relative (*p* = 0.035) heart weights. The second experiment was carried out to determine the effect of AKG on 15-day-old broilers under acute heat stress (AHS) for 3.5 h at 33 ± 1 °C. Forty-eight birds (12 per treatment) were raised until 15 days of age, divided into four treatments with equal numbers (*n* = 12), and given one of the following four treatments: (i) CON group reared at standard temperature (25 ± 1 °C) (CON-NT); (ii) CON group subjected to AHS (33 ± 1 °C) for 3.5 h (CON-HT); (iii) AL group subjected to AHS (33 ± 1 °C) for 3.5 h (AL-HT); and (iv) AH group subjected to AHS (33 ± 1 °C) for 3.5 h (AH-HT). There was a significant reduction in the change in BW (ΔBW, *p* = 0.005), an increase in the final rectal temperature (RTf) (*p* = 0.001), and a decreased final body weight (BWf) for all the treatments under AHS. Further, AHS led to an increased expression of hepatic heat shock protein (HSP)70 (*p* = 0.009), nicotinamide adenine dinucleotide phosphate hydrogen oxidase (NOX)1 (*p* = 0.006), and NOX4 (*p* = 0.001), while nuclear factor erythroid 2-related factor (NRF2), superoxide dismutase (SOD), catalase (CAT), and glutathione peroxidase 1 (GPX1) remained significantly unaffected. Hepatic expression of HSP90 decreased in the AL-HT treatment as compared to CON-HT (*p* = 0.008). Plasma antioxidant status measured by malondialdehyde (MDA) concentration and antioxidant balance (AB) improved linearly (*p* = 0.001) as the concentration of AKG increased. Neonatal gavage feeding of AKG could potentially alleviate heat stress in broilers by enhancing plasma antioxidant levels and modulating HSP90 expression in the liver.

## 1. Introduction

A fertilized egg, encapsulated as the sole nutrient source, supports the developing embryo under standard incubation protocols. During the final three days of chick embryogenesis, a surge in metabolic activity necessitates enhanced gaseous exchange, meeting the elevated oxygen (O_2_) demands [1]. A high metabolic rate is accompanied by increased production of reactive oxygen species (ROS) in the egg [2]. Yolk lipids produce a high amount of polyunsaturated fatty acids (PUFA) during the later phase of incubation, which are highly susceptible to oxidative deterioration via these ROS [3]. During this critical period, antioxidants are transported from the yolk to the embryo, accumulating in the hepatic tissues in preparation for hatching [4]. These antioxidants play crucial roles in mitigating the harmful effects of ROS generated during metabolic processes and environmental stressors, such as elevated temperatures [5].

ROS are byproducts of normal metabolic processes within the body [6]. When produced in excess, ROS can instigate lipid peroxidation, disrupt cellular membranes, and degrade nucleic acids [7]. The cellular redox status is maintained through a finely tuned oxidative defense mechanism. Central to this defense are key enzymes such as nuclear factor erythroid 2-related factor (NRF2), superoxide dismutase (SOD), catalase (CAT), and glutathione peroxidase 1 (GPX1) [8]. These enzymes orchestrate a concerted effort to neutralize free radicals and restore cellular oxidative balance. However, there are instances when the body’s defenses are overwhelmed by the sheer volume of ROS, leading to a state of oxidative stress [9].

Heat stress (HS) is a known progenitor of oxidative stress in broilers [10]. Anatomically, birds lack sweat glands, consequently minimizing their ability to resist high-temperature conditions [11,12]. HS can detrimentally affect poultry performance at any stage of their development. The effects of acute heat stress (AHS) on broilers as early as 10 days of age have been studied [5]. Previous studies have reported the detrimental effect of HS on growth [13,14], cecal microflora [14,15,16], survivability [17], and the expression of heat shock proteins (HSPs) and antioxidant-related genes [18]. The production and release of HSPs represent a vital cellular response to stressful conditions. HSPs are intricately connected to the physiological state of an organism, making them sensitive indicators of stress. These proteins are known to confer cellular protection during heat stress conditions in chickens [19]. Studies have demonstrated that the expression of HSPs is modulated during acute thermal stress [20,21,22]. Therefore, assessing these gene activities could provide insightful predictions about the broilers’ ability to cope with stress. The aforementioned parameters can be partially or fully restored if birds receive appropriate nutritional interventions [23,24,25].

Early nutrition strategies such as in ovo supplementation and gavage feeding have shown promising results in developing heat resistance [26] and altering gut functions [27], respectively. While early post-hatch feeding has been shown to improve gut health [28] and antibody levels in older broilers [29], delayed early-life nutrition led to reduced body weight (BW) in growing broilers up to 21 days of age, accompanied by higher fear stress during transportation at 30 days [30]. Therefore, early post-hatch nutrition could be a key regulator of growth and production in broilers.

α-ketoglutaric acid (AKG) is a crucial partaker in the citric acid cycle [31]. It readily converts to glutamine in the body. Glutamine is classified as a semi-essential amino acid during stress conditions, despite being non-essential in a normal physiological state [32]. Perinatal feeding of AKG via in ovo injection improved the plasma antioxidant status of broilers at hatch and hepatic gene expression of antioxidant enzymes at day 21 of age [33]. In other poultry species, such as Cherry Valley ducks, dietary supplementation of AKG (0.5%, 1.0%, and 1.5%) during days 1–22 of age improved the anti-oxidative capacity and hepatic and intestinal energy levels [34]. Moreover, supplementation with AKG attenuated the lipopolysaccharide-induced splenic oxidative damage in piglets [35]. In piglet models subjected to hydrogen peroxide-induced oxidative stress, AKG modulated antioxidant-related enzymes such as SOD1 and SOD2, thereby enhancing cellular respiration and antioxidant capacity [36]. In hybrid sturgeon fish under ammonia stress, supplementation with 1% AKG led to increased activity of the antioxidant enzymes SOD and GPX in serum, gills, and intestines and hepatic expression of HSP70 and HSP90 [37]. Hence, AKG was able to mitigate oxidative stress in a variety of species of animals.

Currently, there are limited individual studies on heat stress (HS), gavage feeding, and alpha-ketoglutaric acid (AKG) supplementation in broilers. Therefore, this study was designed as a comprehensive investigation into the effects of AKG gavage feeding on neonatal broiler chicks, particularly focusing on their response to AHS at an early age.

## 2. Materials and Methods

The present study was conducted at the animal research facility of Gyeongsang National University, Korea. All experimental procedures were approved by the Institutional Animal Care and Use Committee of the Gyeongsang National University (GNU-200916-C0058).

### 2.1. Bird Rearing and Group Description

#### 2.1.1. Experiment 1

Seventy-two newly hatched Ross 308 broiler chicks were divided into four treatment groups with equal numbers (*n* = 18) of similar body weights (43 ± 2 g). The treatments were described based on the concentration of AKG administered via gavage. Commercially available AKG (#75890, Sigma-Aldrich, St. Louis, MO, USA) was dissolved in DDW to prepare the fresh solutions of the desired concentrations for gavage feeding. The treatments were (i) CON: chicks administered with 0.6 mL of distilled water (DDW) × 2 groups; (ii) AL: chicks administered with 0.6 mL of 0.1% AKG; and (iii) AH: chicks administered with 0.6 mL of 0.2% AKG.

Twenty-four hours after the gavage feeding, six birds from three treatment groups (CON, AL, and AH) were slaughtered using a carbon dioxide chamber to study organ development and plasma antioxidant capacity. A total of six birds from the second CON treatment were randomly removed to equalize the stocking density in all the cages. The remaining birds were further reared for experiment 2, as described in the next section.

#### 2.1.2. Experiment 2

The birds were reared in battery cages as per standard temperature and humidity guidelines until 2 weeks of age. The brooding temperature was 36 ± 1 °C with 50 ± 5% relative humidity (RH) on the day of hatch. The temperature was gradually reduced to reach 25 ± 1 °C on day 15 of age. A commercial broiler starter ration was offered for the first week, followed by a grower ration for the next week (Nonghyup Feed, Gyeongju, Republic of Korea, Supplementary Table S1). Feed and water were provided ad libitum. A 23 h light + 1 h dark regime was followed for the rearing period.

A total of forty-eight birds were divided into four treatments with equal numbers (*n* = 12). The treatments were described based on the concentration of AKG administered via gavage in experiment 1 and the temperature they were subjected to on the 15th day of rearing. AKG solutions were prepared similarly to those described in experiment 1. The treatments were (i) CON-NT: chicks administered with 0.6 mL DDW for 4 days reared at standard temperature (25 ± 1 °C) on day 15; (ii) CON-HS: chicks administered with 0.6 mL DDW for 4 days subjected to AHS temperature (33 ± 1 °C, 3.5 h) on day 15; (iii) AL-HS: chicks administered with 0.6 mL of 0.1%, 0.2%, 0.3%, and 0.4% AKG solutions on 4 consecutive days after hatch and subjected to AHS temperature (33 ± 1 °C, 3.5 h) on day 15; and (iv) AH-HS: chicks administered with 0.6 mL of 0.2%, 0.4%, 0.6%, and 0.8% AKG solutions on 4 consecutive days after hatch and subjected to AHS temperature (33 ± 1 °C, 3.5 h) on day 15.

**Figure 1:**
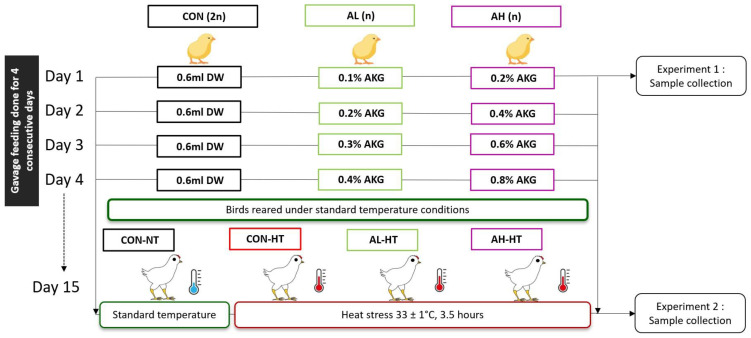
A graphical abstract showing the experimental flow of the present study. Seventy-two newly hatched Ross 308 broiler chicks were divided into four treatment groups with equal numbers (*n* = 18) of similar body weights (43 ± 2 g). In the first experiment, chicks were administered AKG via gavage as (i) CON: chicks administered with 0.6 mL of distilled water (DDW); (ii) AL: chicks administered with 0.6 mL of 0.1% AKG; and (iii) AH: chicks administered with 0.6 mL of 0.2% AKG. Twenty-four hours after gavage feeding, six birds per treatment from three treatment groups (CON, AL, and AH) were slaughtered to study organ development and plasma antioxidant capacity. For experiment 2, forty-eight birds were administered AKG via gavage for 4 days and subjected to AHS on day 15 of age. The treatments were (i) CON-NT: chicks administered with 0.6 mL DDW for 4 days reared at standard temperature (25 ± 1 °C) on day 15; (ii) CON-HS: chicks administered with 0.6 mL DDW for 4 days subjected to AHS temperature (33 ± 1 °C, 3.5 h) on day 15; (iii) AL-HS: chicks administered with 0.6 mL of 0.1%, 0.2%, 0.3%, and 0.4% AKG solutions for 4 days subjected to AHS temperature (33 ± 1 °C, 3.5 h) on day 15; and (iv) AH-HS: chicks administered with 0.6 mL of 0.2%, 0.4%, 0.6%, and 0.8% AKG solutions for 4 days subjected to AHS temperature (33 ± 1 °C, 3.5 h) on day 15. Six birds per treatment were weighed and slaughtered at the end of the heat stress. Biological samples (liver, spleen, intestine, and blood) were collected and processed further.

The AHS protocol was followed as previously described [5,18] with slight modifications. The ambient temperature was gradually increased to 33 ± 1 °C in 30 min, followed by holding the temperature for 3.5 h, and finally reducing it back to 25 ± 1 °C in the next 30 min. During the AHS period, no mortality was recorded. However, the birds displayed behavioral adaptations to AHS by flapping and spreading their wings, panting, etc. Six birds per treatment were slaughtered using the carbon dioxide chamber to collect the biological samples. The study design is presented in Figure 1.

### 2.2. Body Weight and Rectal Temperature

Initial body weight (BW) was measured at the beginning of the AHS experiment (BWi), and final body weight was measured right after the completion of the AHS period (BWf). The change in BW (ΔBW) was derived by subtracting BWi from BWf.

Rectal temperature was measured twice: initially before starting AHS (RTi) and at the end after completing (RTf) the AHS experiment. A digital thermometer (Hanna Instruments Inc., Padova, Italy) was inserted 3 cm into the cloaca, and temperature readings were taken once the temperature stabilized.

### 2.3. Biological Samples

For the first and second experiments, organs were excised free after slaughtering, and their weight was recorded. Different organ weights (experiment 1: liver, yolk sac, intestine, gizzard, proventriculus, and heart; experiment 2: liver, spleen, bursa of Fabricius, gizzard, proventriculus, and heart) were measured. The gizzard was made free of any residual feed before weighing. Blood samples from both experiments were processed in a similar fashion. A total of 2 mL of blood was drawn from the heart before weighing. The amount of blood collected was standardized across all samples to minimize errors in the measurement of heart weight. The samples were transferred to a heparinized vacuum container (#367874, BD Co., Ltd., Franklin Lakes, NJ, USA) to prevent clotting. The tubes were then centrifuged at 2000× *g* for 10 min at 4 °C to separate the plasma. The collected plasma was stored at −20 °C for subsequent processing.

After the AHS experiment, liver samples were collected and processed further. Samples were collected, labeled, and stored in tissue cassettes (#KA-2925-04-CS, Pom tissue embedding deep-cassette, Kartell, Noviglio, Italy). They were immediately snap-frozen by placing them into a liquid nitrogen container.

### 2.4. Plasma Antioxidant Status

The total antioxidant capacity of plasma was measured using the 2,2-diphenyl-1-picrylhydrazyl–radical scavenging activity assay (DPPH-RSA%) as previously outlined [13,33]. Briefly, 20 µL of plasma samples were diluted with 480 µL sodium-potassium phosphate buffer in Eppendorf tubes. An equal volume of 0.1 mmol/L DPPH solution was added to each tube. A control sample was prepared using reagents except for plasma. After incubating the tubes in the dark for 30 min, they were centrifuged at 10,000× *g* for 6 min at 4 °C. The absorbance was then measured at 517 nm, and the inhibitory activity was calculated as a percentage:

DPPH-RSA (%) inhibition = [1 − (A1/A0)] × 100,

where, A0: absorbance of control, A1: absorbance of test samples.

The concentration of malondialdehyde (MDA) in plasma, a marker of lipid peroxidation, was estimated using a protocol adapted from Jyothi et al. [38]. Plasma samples were mixed with 40% trichloroacetic acid (TCA) and 0.67% thiobarbituric acid (TBA) in a 1:1:2 ratio in tubes. The mixture was then incubated in a water bath at 95 °C for 45 min, followed by cooling on ice for 5 min. After centrifugation at 10,000× *g* for 6 min at 4 °C, the absorbance of the supernatant was measured at 530 nm. Plasma MDA concentration was calculated as follows:

MDA concentration (mol/L) = A/(K × h)

where, A: absorbance of sample, K: molar extinction coefficient (1.5 × 10^5^), h: length of the cuvette used (1 cm).

Antioxidant balance (AB) was calculated as the ratio of DPPH-RSA (%) to MDA content following previously described methods [13].

### 2.5. mRNA Quantification Using Real-Time Polymerase Chain Reaction

Liver tissue (50 mg) was homogenized in Trizol™ reagent (# 15596018, Thermo Fisher Scientific, Waltham, MA, USA) and processed for total RNA extraction following methods previously described [18,39]. The concentration and purity of the extracted RNA were assessed using a NanoDrop™ 2000 Spectrophotometer (pedestal mode, Thermo Scientific, Waltham, MA, USA). Subsequently, cDNA synthesis was performed using a commercial kit (Verso cDNA Synthesis Kit # AB1453A, Thermo Fisher, Waltham, MA, USA) according to the manufacturer’s instructions.

Genes of interest were amplified using a StepOnePlus real-time PCR system (Life Technologies, Carlsbad, CA, USA) with a reaction volume of 20 µL. Each reaction contained forward and reverse primers (10 pmol) specific to the genes and 10 µL Power SYBR™ green PCR master mix (# 4312704, Life Technologies, Carlsbad, CA, USA). Ct values of housekeeping genes GAPDH and β-actin were used for normalization of target gene quantification. Fold change (FC) was determined using the 2^−ΔΔCt^ algorithm as previously described [40]. Table 1 represents the primer sequence of the genes studied.

### 2.6. Statistical Analysis

The data obtained in the current study was analyzed using a one-way ANOVA followed by Tukey’s post-hoc test to determine differences between means (*p* < 0.05) in IBM SPSS Statistics for Windows software (IBM SPSS 27; IBM Corp., Armonk, NY, USA). Additionally, polynomial regression analysis was performed to assess dose-dependent effects, presented as linear and quadratic regressions. This analysis excluded the CON-NT treatment from the AHS trial. Before applying appropriate parametric tests, the normality of distribution and homoscedasticity assumptions were evaluated using the Shapiro–Wilk and Levene’s tests, respectively.

For the AHS study, the results for hepatic mRNA expression were categorized into three major groups: (i) Antioxidant-related genes (NRF2, CAT, SOD, GPX1); (ii) Heat shock proteins (HSP70, HSP90); and (iii) NOX-related genes (NOX1, NOX4) for further evaluation through one-way multivariate analysis of variance (MANOVA) using SAS software version 9.4 (SAS Institute Inc., Cary, NC, USA, 2009). The MANOVA was executed using the “manova statement”. Further, “contrast statement” was used to perform planned contrasts and compare: CON vs. CON-HT; CON-HT vs. AL-HT; CON-HT vs. AH-HT; and CON-HT vs. AL-HT and AH-HT.

Karl Pearson’s correlation method was employed to investigate potential inter-relationships among the various parameters analyzed in the AHS study. The statistical analysis was carried out in IBM SPSS Statistics for Windows software (IBM SPSS 27; IBM Corp., Armonk, NY, USA), and the heat map was generated using GraphPad Prism 8 (GraphPad Software, Inc., La Jolla, CA, USA).

## 3. Results

### 3.1. Experiment 1

#### 3.1.1. Organ Weights

Table 2 presents the absolute and relative weights of different organs 24 h after gavage feeding. The absolute weight of the heart was significantly increased (*p* = 0.012) in the AH group, while both the AL and AH groups exhibited a higher gizzard weight in comparison to CON (*p* = 0.015). In terms of relative organ weights, the AL group showed significantly higher gizzard weight (*p* = 0.037), whereas the AH group showed an increased heart weight (*p* = 0.035) compared to CON. Additionally, a linear increase was observed in both the absolute (*p* = 0.020) and relative weights (*p* = 0.040) of the gizzard.

#### 3.1.2. Plasma Antioxidant Capacity

**Figure 2:**
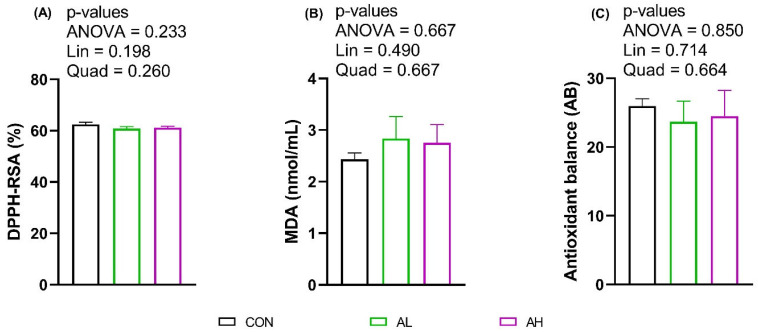
Effect of post-hatch gavage feeding of AKG on the plasma antioxidative parameters, DPPH-RSA (%) (**A**), MDA concentration (nmol/mL) (**B**), and antioxidant balance (**C**) of chicks after 24 h. After hatching, chicks were administered AKG via gavage. The treatments were (i) CON: chicks administered with 0.6 mL of distilled water (DDW); (ii) AL: chicks administered with 0.6 mL of 0.1% AKG; and (iii) AH: chicks administered with 0.6 mL of 0.2% AKG. Data are presented as Mean ± SEM (*n* = 6). Means were analyzed by one-way ANOVA and Tukey’s post-hoc test. Abbreviations: DPPH-RSA (%), 2,2-diphenyl-1-picrylhydrazyl free radical scavenging activity; MDA, malondialdehyde; AB, antioxidant balance; Lin, linear effect; Quad, quadratic effect.

The plasma antioxidant capacity, measured as (DPPH-RSA %), MDA concentration, and AB, were not significantly different among treatments (Figure 2).

### 3.2. Experiment 2

#### 3.2.1. Body Weight and Organ Indices

Initial (BWi) and final body weights (BWf) did not differ significantly across treatments. However, the change in BW (ΔBW) differed significantly (Table 3). A decrease in BW was measured in all the groups subjected to AHS, while CON-NT treatment showed an increase in BW (*p* = 0.005).

Table 4 presents different absolute and relative organ weights (liver, spleen, bursa of Fabricius, proventriculus, gizzard, and heart), which did not differ significantly across treatments.

#### 3.2.2. Rectal Temperature

The initial rectal temperature (RTi) measured before commencing the AHS experiment did not differ significantly among treatment groups (Table 5). However, after the AHS experiment was concluded, all groups subjected to AHS exhibited a significantly higher final rectal temperature (RTf) compared to CON-NT (*p* = 0.001). Similarly, the change in RT (ΔRT) was significantly higher in all AHS treatments compared to CON-NT (*p* = 0.002).

#### 3.2.3. Plasma Antioxidant Capacity

**Figure 3:**
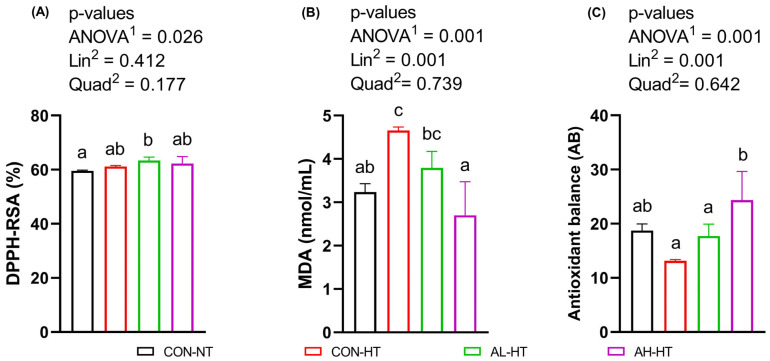
Effect of AKG gavage feeding for four consecutive days on plasma antioxidant parameters, DPPH-RSA (%) (**A**), MDA concentration (nmol/mL) (**B**), and antioxidant balance (**C**) of broiler chicks subjected to AHS at day 15 of age. After hatching, chicks were administered AKG via gavage. The treatments were (i) CON-NT: chicks administered with 0.6 mL DDW for 4 days reared at standard temperature (25 ± 1 °C) on day 15; (ii) CON-HS: chicks administered with 0.6 mL DDW for 4 days subjected to AHS temperature (33 ± 1 °C, 3.5 h) on day 15; (iii) AL-HS: chicks administered with 0.6 mL of 0.1%, 0.2%, 0.3%, and 0.4% AKG solutions for 4 days subjected to AHS temperature (33 ± 1 °C, 3.5 h) on day 15; and (iv) AH-HS: chicks administered with 0.6 mL of 0.2%, 0.4%, 0.6%, and 0.8% AKG solutions for 4 days subjected to AHS temperature (33 ± 1 °C, 3.5 h) on day 15. Data are presented as Mean ± SEM (*n* = 6). Means were analyzed by one-way ANOVA and Tukey’s post-hoc test. ^a–c^ Means bearing different letters indicate a statistical difference (*p* < 0.05). ^1^ *p*-value of all treatment groups. ^2^ *p*-value of all treatment groups except CON-NT. Abbreviations: DPPH-RSA (%), 2,2-diphenyl-1-picrylhydrazyl free radical scavenging activity; MDA, malondialdehyde; AB, antioxidant balance; Lin, linear effect; Quad, quadratic effect.

Figure 3 illustrates that the AL-HT group displayed higher free radical scavenging activity (DPPH-RSA) as compared to CON-NT (*p* = 0.026). Furthermore, the plasma MDA concentration was significantly lower in the AH-HT group as compared to the CON-HT group (*p* = 0.001). There was a significant linear decrease (*p* = 0.01) in plasma MDA concentration. Concurrently, plasma AB showed improvement, with the highest levels observed in AH-HT compared to CON-HT and AL-HT (*p* = 0.001).

#### 3.2.4. Hepatic Gene Expression of Antioxidant-Related Gens

**Figure 4:**
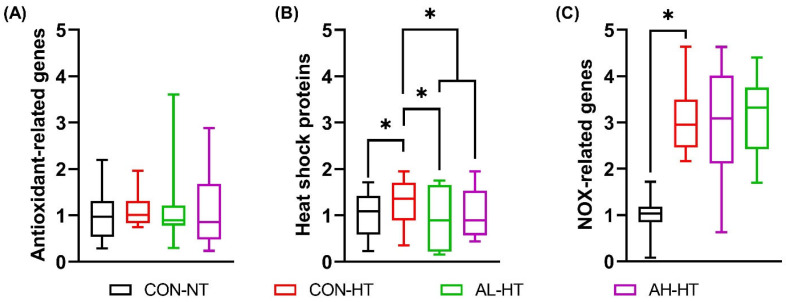
Boxplots showing the assessment of the effects of AKG gavage feeding for four consecutive days on hepatic mRNA expression of a set of (**A**) antioxidant-related genes, (**B**) heat shock proteins, and (**C**) NOX-related genes of broiler chicks subjected to AHS at day 15 of age using MANOVA. After hatching, chicks were administered AKG via gavage. The treatments were (i) CON-NT: chicks administered with 0.6 mL DDW for 4 days reared at standard temperature (25 ± 1 °C) on day 15; (ii) CON-HS: chicks administered with 0.6 mL DDW for 4 days subjected to AHS temperature (33 ± 1 °C, 3.5 h) on day 15; (iii) AL-HS: chicks administered with 0.6 mL of 0.1%, 0.2%, 0.3%, and 0.4% AKG solutions for 4 days subjected to AHS temperature (33 ± 1 °C, 3.5 h) on day 15; and (iv) AH-HS: chicks administered with 0.6 mL of 0.2%, 0.4%, 0.6%, and 0.8% AKG solutions for 4 days subjected to AHS temperature (33 ± 1 °C, 3.5 h) on day 15. * Means bearing different symbols indicate a statistical difference (*p* < 0.05).

The overall MANOVA (Figure 4A) and contrast results (Table 6) indicated no significant changes in antioxidant-related gene expression in the liver. Similarly, hepatic expression of antioxidant-related enzymes, NRF2, CAT, SOD, and GPX1, did not show significant differences among treatments (Table 7).

#### 3.2.5. Hepatic Gene Expression of Heat Shock Proteins

Table 7 displays the expression of HSP70 and HSP90 in the liver. HSP70 followed a similar expressional pattern to NOX4, where all the treatments subjected to AHS showed a significantly higher expression (*p* = 0.009) as compared to CON-NT.

Interestingly, HSP90 did not show a similar pattern of expression and was expressed significantly lower (*p* = 0.008) in the AL-HT group as compared to CON-NT. Furthermore, a significant quadratic increase (*p* = 0.003) in HSP90 was seen with an increasing dose of AKG gavage under AHS.

The overall MANOVA of HSP genes showed significant differences (*p* < 0.001), as shown in Table 6. AHS led to an overall increase in the expression of HSPs in contrast to CON-NT (CON-NT vs. CON-HT, *p* = 0.006). However, in contrast to CON-HT, AL-HT showed a significant reduction in the expression of HSPs (*p* = 0.012). Putting together, AKG gavage (AL-HT and AH-HT) groups vs. CON-HT led to an overall decrease (*p* = 0.036) in the expression of HSPs (Figure 4B).

#### 3.2.6. Hepatic Gene Expression of NOX-Related Genes

NOX-related genes and genes related to ROS production were also studied (Table 7). CON-HT and AL-HT showed significantly higher NOX1 mRNA expression than CON-NT (*p* = 0.006). NOX4 expression was also significantly higher in all AHS-treated groups compared to CON-NT (*p* = 0.001).

An overall upregulation of NOX-related genes (*p* < 0.001, MANOVA) in the CON-HT treatment was estimated relative to the CON-NT group (Figure 4C). The expression of NOXs remained unaffected by the gavage feeding of AKG to broilers under AHS (Table 6).

#### 3.2.7. Interrelationship between Core Body Temperature, Growth, Hepatic Gene Expression of Various Sets of Genes, and Plasma Antioxidant Capacity

**Figure 5:**
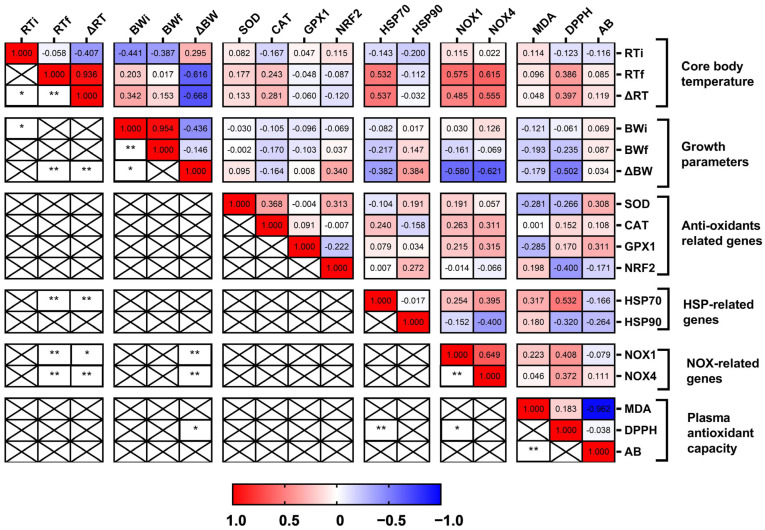
Pearson’s correlation plot among different parameters studied during the AHS study at 15 days of age. The parameters are divided into “Core body temperature”, “Growth parameters”, “Antioxidant-related genes”, “HSP-related genes”, “NOX-related genes”, and “Plasma antioxidant capacity”. The data were analyzed using IBM SPSS Statistics for Windows software (IBM SPSS 27; IBM Corp., Armonk, NY, USA). A heat map was realized using GraphPad Prism 8 (GraphPad Software, Inc., La Jolla, CA, USA). Red indicates a positive correlation, blue indicates a negative correlation, and white indicates no correlation. The r values are indicated in the graph. X indicates a non-significant correlation. * indicates significant correlation at *p* < 0.05 level. ** indicates significant correlation at *p* < 0.01 level.

A Pearson’s correlation analysis was executed to explore possible relationships between various parameters obtained in the study (Figure 5). Core body temperature showed various significant correlations with other parameters in this study. ΔRT was significantly correlated with RTi (r = −0.407, *p* < 0.01) and RTf (r = 0.936, *p* < 0.05). A negative correlation between RTi and BWi was also noticed (r = −0.441, *p* < 0.05). ΔBW was found to be negatively correlated (*p* < 0.01) with the RTf (r = −0.616) and ΔRT (r = −0.668). Further, the hepatic expression of HSP70 was also found to be significantly correlated (*p* < 0.01) with RTf (r = 0.532) and ΔRT (r = 0.537). A significant positive correlation (*p* < 0.01) between the expression of NOX1 (r = 0.575) and NOX4 (r = 0.615) with RTf was seen. ΔRT was also found to be positively correlated with the expression of NOX1 (r = 0.485, *p* < 0.05) and NOX4 (r = 0.555, *p* < 0.001). The initial BW (BWi) was positively correlated with BWf (r = 0.954, *p* < 0.01) but negatively correlated with ΔBW (r = −0.436, *p* < 0.05). Moreover, ΔBW was found to be negatively correlated with the expression of NOX1 (r = −0.580, *p* < 0.01) and NOX4 (r = −0.621, *p* < 0.01). The expression of NOX1 and NOX4 was positively correlated among themselves (r = 0.649, *p* < 0.01). DPPH-RSA (%), a plasma antioxidant marker, was negatively correlated with the ΔBW (r = −0.502, *p* < 0.05) while being positively correlated with the expression of HSP70 (r = 0.532, *p* < 0.01) and NOX1 (r = 0.408, *p* < 0.01). Plasma AB was also significantly negatively correlated with the plasma MDA concentration (r = −0.962, *p* < 0.01).

## 4. Discussion

Early-life nutrition is crucial for broilers’ growth and development. Precisely, a newly hatched chick transitions from relying on the chorioallantoic membrane for gaseous exchange during prenatal life to pulmonary respiration facilitated by the lungs during postnatal life [41]. The quality of day-old chicks and early-life nutrition are highly linked with broiler production. In fact, the relative development of digestive organs such as the proventriculus, gizzard, and small intestine supersedes the development of other organs during the early post-hatch period [42]. To the authors’ knowledge, no literature exists on the gavage feeding of AKG to newly hatched broilers. Therefore, birds were slaughtered 24 h after the gavage feeding to study its effects on organ development. Both gizzard weight and heart indices were significantly higher in the AH treatments compared to CON and showed a linear increase. As broilers continue to be selected over the years for superior growth performance, a reduction in overall heart size has been witnessed [43]. The study concluded that if the observed trends persist, the potential damage caused by decreasing heart sizes will worsen over time. A study conducted by Geng et al. (2004) aimed at reducing mortality caused by ascites in broilers showed that supplementation of Coenzyme Q10 at a 20 mg/kg diet increased the heart weight of the broilers [44]. Upon further analysis of various other parameters, they concluded that a 40 mg/kg inclusion of Coenzyme Q10 in the diet was a better dose to alleviate the mortality caused by ascites in broilers. The current findings align with our previous study, where ovo feeding of 0.5% AKG at 0.6 mL per egg significantly increased the heart indices of broilers [33]. Additionally, in human patients, AKG administered during blood cardioplegia improved myocardial protection [45]. AKG has been demonstrated to mitigate cardiac dysfunction induced by pressure overload in mice [46]. Supplementation of 2% AKG in mice was found to protect stress-overloaded hearts [46]. The myocardial protective effect is attributed to the antioxidative functions of AKG. AKG helped eliminate damaged mitochondria in heart muscles, thereby reducing ROS production [47]. AKG is known to metabolize in the heart muscles of pigeons to produce glutamic acid [48]. Nevertheless, AKG is a key component of the Krebs cycle in the body, contributing to energy production [49]. In broilers, high metabolic rates drive high oxygen demand [50]. However, due to a lack of analogous development of the cardio-respiratory system, cells suffer from hypoxia. In the current study, the observed improvement in heart size after the initial gavage feeding of AKG may indicate positive cardio-vascular development in broilers. These cardioprotective effects of AKG are likely due to its ability to reduce ROS generation and could be further investigated as a potential strategy to mitigate losses from ascites or other cardiac-related disorders.

AHS is known to negatively impact the BWG of broilers [14,15]. During high ambient temperatures, birds exhibit panting behavior [51]. This reduces feed intake and correlates with decreased growth performance in broilers. Previous research, including our own, found that ovo feeding of AKG did not improve BW in broilers under cyclic heat stress [26]. Similar results were reported by Tomaszewska et al. (2020), where dietary AKG supplementation did not affect BW in laying hens reared under thermoneutral conditions [52]. Under standard rearing conditions, in ovo feeding of AKG did not improve or deter growth performance in broilers up to 3 weeks of age [33]. In mirror carps, supplementation of AKG in a low-phosphorous diet did not improve growth performance [53]. Conversely, grass carp supplemented with 7.5 g/kg AKG showed an increase in body weight and improved immune status [54]. Similarly, piglets fed a low-protein diet supplemented with 0.5%, 1.0%, or 1.5% AKG showed an improved final BW in a 28-day trial period [55]. These findings indicate that the effects of AKG on growth performance vary across different animal species, ages, and dietary treatments. AKG is efficiently converted to glutamine in muscles [48]. In enterocytes, glutamine serves as an energy source [35], promoting intestinal development [56]. It could potentially enhance nutrient absorption from the intestine, leading to improved growth [56]. In the present study, parameters related to intestinal development, such as villi height and crypt depth, were not assessed. Future studies focusing on these parameters could provide further insights. However, in the current study, AHS conditions appeared to hinder growth, and gavage feeding of AKG did not alleviate the adverse effects of AHS on the BW of the broilers at 15 days of age.

Core body temperature is a reliable indicator to visualize the effects of ambient temperature on broilers. Birds, as homeotherms, can sustain the core temperature irrespective of fluctuations in ambient temperature [57]. However, studies have shown that RT increases with an increase in ambient temperature [13,17,58]. RT has also been negatively correlated with BWG in broilers [59], which supports the decreased BWG observed in our study under AHS. All the treatments subjected to AHS showed higher RT and a loss in BW. Our previous study on broiler chickens subjected to cyclic heat stress during days 28–34 showed that in ovo feeding of AKG enfeebled the increase in RT but could not prevent it [26]. This discrepancy in result might be due to different modes of feeding (in ovo vs. gavage), the age of birds, and the duration of heat stress. Therefore, AKG gavage might not strongly regulate RT in broilers during AHS; instead, ambient temperature negatively affects the RT of the birds significantly.

HS is overtly capable of eliciting oxidative stress in broilers [10]. During HS, NRF2 plays a vital role [60] by activating the machinery of the major antioxidant enzymes, namely, CAT, SOD, and GPX1 [33]. However, in the current study, these antioxidant enzymes showed no significant changes. Disturbance in the cellular oxidation state causes excessive generation and accumulation of free radicals in the body [61]. Dysfunctional oxidative processes can elevate the expression of NOX enzymes, which contribute to an unfavorable redox balance by transferring electrons from NADPH to oxygen [62]. This disrupts the normal functioning of the cells, leading to cell death [63]. In a previous study, in ovo feeding of AKG reduced the expression of hepatic NOX1 and NOX4 in broilers under cyclic HS [26]. However, in the current study, AKG gavage did not downregulate the expression of hepatic NOX1 and NOX4 enzymes. All the treatments under AHS elicited a higher expression of NOX-related genes. Contrast analysis further supported these findings, showing significantly higher expression in CON-HT compared to CON-NT. Additionally, the correlation matrix revealed that NOX enzymes were positively correlated with the final RT and ΔRT while negatively correlated with the ΔBW. This suggests that high levels of ROS adversely affect production performance in broilers. This is due to the ability of ROS to cause irreversible damage to macromolecules such as proteins, lipids, and carbohydrates in the body, triggered via cell apoptosis [64]. This causes a deterioration in meat quality and quantity. AKG helps to scavenge free radicals via enzymatic and non-enzymatic pathways [36]. However, its precise effect on the regulation of enzymes like NOXs is not well understood. Further studies are needed to elucidate the molecular pathways through which AKG affects ROS-producing enzymes such as NOXs in broilers.

HSPs, such as HSP70 and HSP90, are key indicators of thermotolerance in broilers under HS [22]. In the current study, AHS increased the expression of HSP70 regardless of AKG treatment. HSPs act as molecular chaperones that are highly expressed during stress conditions [65]. For instance, HSP70 is upregulated due to the stimulation of hepatocytes via thermal stimuli [66]. Contrast analysis confirmed higher expression of HSPs during AHS, reflecting their role in thermal stress. Furthermore, a positive correlation between the expression of HSP70 and RTf also underscores their involvement in the response to thermal stress. These findings align with previous studies where increased expression of HSP70 [67] and HSP90 [68] was observed under HS conditions. However, in the current study, gavage feeding of AKG appeared to downregulate HSP expression, supported by MANOVA results. Conversely, 1.0% AKG increased the expression of HSP70 and HSP90 in hybrid sturgeons under ammonia stress [37]. Similarly, 1.0% AKG prevented intestinal upregulation of HSP70 in lipopolysaccharide (LPS)-challenged piglets [26]. Nonetheless, dietary AKG at varied levels from 0.25% to 1.0% did not affect the hepatopancreatic expression of HSP70 [69]. In our previous study, ovo feeding of AKG decreased the expression of HSPs under cyclic heat stress in broilers [26]. These variations suggest that HSP expression might vary depending on species, environmental conditions, age, dosage, and methods of supplementation. Further studies are needed to clarify the specific effects of gavage feeding of AKG on HSP expression in broilers under HS conditions.

HS impairs the ability of organisms to counteract ROS, leading to cellular membrane damage and increased production of MDA, a marker of lipid oxidation [70]. In this study, MDA levels were elevated in the birds under AHS. However, AKG gavage reduced MDA concentrations in a dose-dependent manner, with higher AKG concentrations leading to a greater reduction in MDA. This decline in MDA was accompanied by a linear rise in AB in the birds. Similar findings were observed in rats with induced ammonium toxicity, where 2 g/kg BW of AKG supplementation significantly reduced lipid peroxidation [71]. The same study demonstrated that AKG participated in non-enzymatic hydrogen peroxide decomposition and ammonia detoxification. Additionally, calcium-AKG supplementation in aged mice reduced plasma thiobarbituric acid reactive substances (TBARS) levels and improved the total antioxidant [72]. In the current study, the free radical scavenging activity of plasma, measured as DPPH-RSA (%) activity, improved in the AL-HT group as compared to CON-NT. Previous studies support these results, showing that in ovo feeding of AKG can increase the DPPH-RSA activity at hatch [33] and improve plasma antioxidant status during cyclic HS in broilers [26]. Hence, gavage feeding of AKG helps alleviate the plasma oxidative stress caused by AHS in broilers.

The study design had certain shortcomings and, thus, some limitations in the interpretation. First, the repetitive and graded nature of AKGs gavage administration limited the number of birds used in this study, resulting in fewer birds used for feeding and HS studies. Thus, the effects of neonatal AKG feeding on feed intake and growth remain to be tested in broilers. Second, in the current study, AKG was administered over four days via gavage in increasing amounts. A single, one-time gavage might have different effects than multiple gavages, even if the total amount administered is the same. Third, the study lacked a negative control group. Gavage feeding itself can be a stressful event. Therefore, a control group should be included to filter out the effects of gavage itself in the next study.

## 5. Conclusions

Gavage feeding of AKG did not influence body weight in broilers under AHS at 15 days of age. While there were no significant effects on the hepatic expression of antioxidant- and NOX-related enzymes, AKG at low doses (AL-HT) decreased the expression of HSP90 compared to CON-NT and CON-HT. Additionally, RT emerged as an important physiological parameter under AHS, correlating with BW, HSP70, and NOX-related genes. Notably, AKG gavage helped mitigate plasma oxidative stress caused by AHS, as evidenced by regression analysis of AB and MDA levels. Thus, perinatal gavage feeding of AKG may help alleviate some adverse effects of AHS by modulating hepatic HSP90 and enhancing blood antioxidant status in young broilers. However, further comprehensive studies are needed to clarify the impact of neonatal AKG feeding on growth and heat stress in broiler chickens.

## Figures and Tables

**Table 1:** Oligonucleotide primer sequence for RT-qPCR.

No.	Gene	Sequence	Accession Number	Reference
1.	GAPDH	F: TTGGCATTGTGGAGGGTCTTA	NM_204305.1	[13]
		R: GTGGACGCTGGGATGATGTT		
2.	β-actin	F: ACCGGACTGTTACCAACA	NM_205518.1	[41]
		R: GACTGCTGCTGACACCTT		
3.	NRF2	F: CAGAAGCTTTCCCGTTCATAGA	NM_205117	[41]
		R: GACATTGGAGGGATGGCTTAT		
4.	CAT	F: ACCAAGTACTGCAAGGCGAA	NM_001031215.1	[41]
		R: TGAGGGTTCCTCTTCTGGCT		
5.	SOD	F: AGGGGGTCATCCACTTCC	NM_205064.1	[42]
		R: CCCATTTGTGTTGTCTCCAA		
6.	GPX1	F: AACCAATTCGGGCACCAG	NM_001277853.2	[5]
		R: CCGTTCACCTCGCACTTCTC		
7.	HSP70	F: GCTGAACAAGAGCATCAATCCA	AY143693.1	[42]
		R: CAGGAGCAGATCTTGCACATTT		
8.	HSP90	F: CCCGAGCAAGCTGGATTCT	NM_001109785	[18]
		R: GGTCATCCCTATGCCGGTATC		
9.	NOX1	F: GCGAAGACGTGTTCCTGTAT	NM_001101830.1	[39]
		R: GAACCTGTACCAGATGGACTTC		
10.	NOX4	F: CCTCTGTGCTTGTACTGTGTAG	NM_001101829.1	[39]
		R: GACATTGGAGGGATGGCTTAT		

Abbreviations: GAPDH, glyceraldehyde-3-phosphate dehydrogenase; β-actin, beta-actin; NRF2, nuclear factor erythroid 2-related factor; CAT, catalase; SOD, superoxide dismutase; GPX1, glutathione peroxidase 1; HSP70, heat shock protein 70; HSP90, heat shock protein 90; NOX1, Nicotinamide adenine dinucleotide phosphate oxidase 1; NOX4, Nicotinamide adenine dinucleotide phosphate oxidase 4.

**Table 2:** Effect of post-hatch gavage feeding of AKG on the organ weights of chicks after 24 h.

Parameters	Treatments			Pooled SEM	*p*-Value		
	CON	AL	AH		ANOVA	Lin	Quad
Absolute organ weight (g)							
Liver	1.823	1.820	1.758	0.05	0.854	0.612	0.799
Yolk sac	2.797	2.183	2.578	0.14	0.201	0.541	0.097
Intestine	4.235	4.695	4.577	0.12	0.301	0.269	0.277
Gizzard	4.160 ^a^	4.895 ^b^	4.833 ^b^	0.12	0.015	0.020	0.077
Proventriculus	0.713	0.748	0.697	0.03	0.804	0.833	0.537
Heart	0.402 ^a^	0.425 ^ab^	0.495 ^b^	0.01	0.012	0.004	0.349
Relative organ weight (g/100 g BW)							
Liver	3.523	3.377	3.267	0.09	0.499	0.232	0.922
Yolk sac	5.433	4.077	4.788	0.28	0.150	0.370	0.087
Intestine	8.171	8.708	8.504	0.18	0.486	0.460	0.347
Gizzard	8.048 ^a^	9.085 ^b^	8.983 ^ab^	0.19	0.037	0.040	0.119
Proventriculus	1.379	1.394	1.291	0.06	0.752	0.544	0.649
Heart	0.779 ^a^	0.790 ^ab^	0.920 ^b^	0.03	0.035	0.021	0.222

After hatching, chicks were administered AKG via gavage. The treatments were (i) CON: chicks administered with 0.6 mL of distilled water (DDW); (ii) AL: chicks administered with 0.6 mL of 0.1% AKG; and (iii) AH: chicks administered with 0.6 mL of 0.2% AKG. Data are presented as Mean ± SEM (*n* = 6). ^a,b^ Means bearing different letters differ significantly in a row (*p* < 0.05). Abbreviations: Lin, linear effect; Quad, quadratic effect.

**Table 3:** Effect of AKG gavage on body weight of broilers subjected to AHS at day 15 of age.

Parameters	Treatments				Pooled SEM	*p* Value		
	CON-NT	CON-HT	AL-HT	AH-HT		ANOVA ^1^	Lin ^2^	Quad ^2^
Initial body weight	521.00	521.33	533.67	536.00	9.33	0.921	0.613	0.846
Final body weight	531.00	513.00	519.67	526.00	8.48	0.903	0.615	0.994
ΔBW	10.00 ^b^	−8.33 ^a^	−14.00 ^a^	−10.00 ^a^	2.83	0.005	0.816	0.442

After hatching, chicks were administered AKG via gavage. The treatments were (i) CON-NT: chicks administered with 0.6 mL DDW for 4 days reared at standard temperature (25 ± 1 °C) on day 15; (ii) CON-HS: chicks administered with 0.6 mL DDW for 4 days subjected to AHS temperature (33 ± 1 °C, 3.5 h) on day 15; (iii) AL-HS: chicks administered with 0.6 mL of 0.1%, 0.2%, 0.3%, and 0.4% AKG solutions for 4 days subjected to AHS temperature (33 ± 1 °C, 3.5 h) on day 15; and (iv) AH-HS: chicks administered with 0.6 mL of 0.2%, 0.4%, 0.6%, and 0.8% AKG solutions for 4 days subjected to AHS temperature (33 ± 1 °C, 3.5 h) on day 15. Data are presented as Mean ± SEM (*n* = 6). ^a,b^ Means bearing different letters differ significantly in a row (*p* < 0.05). ^1^ *p*-value of all treatment groups. ^2^ *p*-value of all treatment groups except CON-NT. Abbreviations: ΔBW, change in body weight; Lin, linear effect; Quad, quadratic effect.

**Table 4:** Effect of AKG gavage on various organ weights (liver, spleen, bursa of Fabricius, proventriculus, gizzard, and heart) of broilers subjected to AHS at day 15 of age.

Parameters	Treatments				Pooled SEM	*p* Value		
	CON-NT	CON-HT	AL-HT	AH-HT		ANOVA ^1^	Lin ^2^	Quad ^2^
Absolute organ weight (g)								
Liver	21.06	22.11	20.42	22.44	0.64	0.691	0.872	0.306
Spleen	0.51	0.47	0.51	0.51	0.02	0.908	0.553	0.821
Bursa of Fabricius	0.98	0.91	0.95	0.92	0.05	0.964	0.958	0.781
Proventriculus	3.17	3.25	3.04	3.21	0.12	0.945	0.902	0.492
Gizzard	8.05	8.94	8.47	7.96	0.22	0.419	0.165	0.976
Heart	4.04	4.08	3.59	4.08	0.14	0.565	0.997	0.187
Relative organ weight (g/100 g BW)								
Liver	3.97	4.32	3.93	4.30	0.13	0.604	0.963	0.290
Spleen	0.10	0.09	0.10	0.10	0.00	0.969	0.696	0.814
Bursa of Fabricius	0.18	0.18	0.18	0.17	0.01	0.979	0.937	0.779
Proventriculus	0.60	0.63	0.59	0.61	0.02	0.883	0.612	0.433
Gizzard	1.52	1.75	1.64	1.54	0.05	0.405	0.205	0.962
Heart	0.76	0.79	0.69	0.78	0.02	0.344	0.816	0.063

After hatching, chicks were administered AKG via gavage. The treatments were (i) CON-NT: chicks administered with 0.6 mL DDW for 4 days reared at standard temperature (25 ± 1 °C) on day 15; (ii) CON-HS: chicks administered with 0.6 mL DDW for 4 days subjected to AHS temperature (33 ± 1 °C, 3.5 h) on day 15; (iii) AL-HS: chicks administered with 0.6 mL of 0.1%, 0.2%, 0.3%, and 0.4% AKG solutions for 4 days subjected to AHS temperature (33 ± 1 °C, 3.5 h) on day 15; and (iv) AH-HS: chicks administered with 0.6 mL of 0.2%, 0.4%, 0.6%, and 0.8% AKG solutions for 4 days subjected to AHS temperature (33 ± 1 °C, 3.5 h) on day 15. Data are presented as Mean ± SEM (*n* = 6). ^1^ *p*-value of all treatment groups. ^2^ *p*-value of all treatment groups except CON-NT. Abbreviations: Lin, linear effect; Quad, quadratic effect.

**Table 5:** Effect of AKG gavage on rectal temperature of broilers subjected to AHS at day 15 of age.

Parameters	Treatments				Pooled SEM	*p* Value		
	CON-NT	CON-HT	AL-HT	AH-HT		ANOVA ^1^	Lin ^2^	Quad ^2^
RTi	41.60	41.67	41.65	41.65	0.05	0.979	0.919	0.955
RTf	41.48 ^a^	42.80 ^b^	42.45 ^b^	42.87 ^b^	0.14	0.001	0.818	0.124
ΔRT	−1.2 ^a^	1.13 ^b^	0.80 ^b^	1.22 ^b^	0.15	0.002	0.822	0.247

After hatching, chicks were administered AKG via gavage. The treatments were (i) CON-NT: chicks administered with 0.6 mL DDW for 4 days reared at standard temperature (25 ± 1 °C) on day 15; (ii) CON-HS: chicks administered with 0.6 mL DDW for 4 days subjected to AHS temperature (33 ± 1 °C, 3.5 h) on day 15; (iii) AL-HS: chicks administered with 0.6 mL of 0.1%, 0.2%, 0.3%, and 0.4% AKG solutions for 4 days subjected to AHS temperature (33 ± 1 °C, 3.5 h) on day 15; and (iv) AH-HS: chicks administered with 0.6 mL of 0.2%, 0.4%, 0.6%, and 0.8% AKG solutions for 4 days subjected to AHS temperature (33 ± 1 °C, 3.5 h) on day 15. Data are presented as Mean ± SEM (*n* = 6). ^a,b^ Means bearing different letters differ significantly in a row (*p* < 0.05). ^1^ *p*-value of all treatment groups. ^2^ *p*-value of all treatment groups except CON-NT. Abbreviations: RTi, rectal temperature initial; RTf, rectal temperature final; ΔRT, change in rectal temperature; Lin, linear effect; Quad, quadratic effect.

**Table 6:** MANOVA and multivariate planned contrasts on different sets of hepatic gene expressions in broilers subjected to AHS at day 15 of age.

Set of Genes	MANOVA	Multivariate Planned Contrast			
		CON-NT vs. CON-HT	CON-HT vs. AL-HT	CON-HT vs. AH-HT	CON-HT vs. AL-HT and AH-HT
Antioxidant-related genes	0.073	0.681	0.193	0.079	0.233
Heat shock proteins	<0.001	0.006	0.012	0.315	0.036
NOX-related genes	<0.001	<0.001	0.108	0.292	0.116

After hatching, chicks were administered AKG via gavage. The treatments were (i) CON-NT: chicks administered with 0.6 mL DDW for 4 days reared at standard temperature (25 ± 1 °C) on day 15; (ii) CON-HS: chicks administered with 0.6 mL DDW for 4 days subjected to AHS temperature (33 ± 1 °C, 3.5 h) on day 15; (iii) AL-HS: chicks administered with 0.6 mL of 0.1%, 0.2%, 0.3%, and 0.4% AKG solutions for 4 days subjected to AHS temperature (33 ± 1 °C, 3.5 h) on day 15; and (iv) AH-HS: chicks administered with 0.6 mL of 0.2%, 0.4%, 0.6%, and 0.8% AKG solutions for 4 days subjected to AHS temperature (33 ± 1 °C, 3.5 h) on day 15. Data are presented as Mean ± SEM (*n* = 6). The Wilks’ Lambda *p*-values are reported. Abbreviations: NOX, Nicotinamide adenine dinucleotide phosphate oxidase; MANOVA, multivariate analysis of variance.

**Table 7:** Effect of AKG gavage feeding on relative hepatic expression of various genes in broilers subjected to AHS at day 15 of age.

Parameters	Treatments				Pooled SEM	*p* Value		
	CON-NT	CON-HS	AL-HS	AH-HS		ANOVA ^1^	Lin ^2^	Quad ^2^
Antioxidant-related genes								
NRF2	1.00	1.20	0.70	0.76	0.09	0.175	0.071	0.167
CAT	1.00	1.36	1.98	1.10	0.16	0.103	0.606	0.073
SOD	1.00	1.01	0.86	1.33	0.13	0.638	0.406	0.359
GPX1	1.00	0.80	0.98	1.31	0.12	0.575	0.139	0.793
NOX-related genes								
NOX1	1.00 ^a^	3.21 ^b^	2.67 ^b^	2.56 ^ab^	0.25	0.006	0.306	0.695
NOX4	1.00 ^a^	2.91 ^b^	3.56 ^b^	3.19 ^b^	0.25	0.001	0.564	0.234
HSP-related genes								
HSP70	1.00 ^a^	1.61 ^b^	1.59 ^b^	1.51 ^b^	0.08	0.009	0.491	0.805
HSP90	1.00 ^b^	0.93 ^b^	0.24 ^a^	0.59 ^ab^	0.10	0.008	0.136	0.003

After hatching, chicks were administered AKG via gavage. The treatments were (i) CON-NT: chicks administered with 0.6 mL DDW for 4 days reared at standard temperature (25 ± 1 °C) on day 15; (ii) CON-HS: chicks administered with 0.6 mL DDW for 4 days subjected to AHS temperature (33 ± 1 °C, 3.5 h) on day 15; (iii) AL-HS: chicks administered with 0.6 mL of 0.1%, 0.2%, 0.3%, and 0.4% AKG solutions for 4 days subjected to AHS temperature (33 ± 1 °C, 3.5 h) on day 15; and (iv) AH-HS: chicks administered with 0.6 mL of 0.2%, 0.4%, 0.6%, and 0.8% AKG solutions for 4 days subjected to AHS temperature (33 ± 1 °C, 3.5 h) on day 15. Data are presented as Mean ± SEM (*n* = 6). Means were analyzed by one-way ANOVA and Tukey’s post-hoc test. ^a,b^ Means bearing different letters indicate a statistical difference (*p* < 0.05). ^1^ *p*-value of all treatment groups. ^2^ *p*-value of all treatment groups except CON-NT. Abbreviations: NRF2, nuclear factor erythroid 2–related factor; SOD, superoxide dismutase; CAT, catalase; GPX1, glutathione peroxidase 1; NOX1, Nicotinamide adenine dinucleotide phosphate oxidase 1; NOX4, Nicotinamide adenine dinucleotide phosphate oxidase 4; HSP70, heat shock protein-70; HSP90, heat shock protein-90; Lin, linear effect; Quad, quadratic effect.

## Data Availability

The original contributions presented in the study are included in the article/Supplementary Materials, further inquiries can be directed to the corresponding author.

## Supplementary Materials

The following supporting information can be downloaded at: https://www.mdpi.com/article/10.3390/ani14152243/s1, Table S1: Feed composition and nutrient levels of the diet *.
