# Supplementary material for: Neonatal α-Ketoglutaric Acid Gavage May Potentially Alleviate Acute Heat Stress by Modulating Hepatic Heat Shock Protein 90 and Improving Blood Antioxidant Status of Broilers
Source: Animals (Basel). 2024 Aug 1;14(15):2243. doi: 10.3390/ani14152243 (PMC11310969; doi:10.3390/ani14152243)
Supplement: Supplementary file 1 [file animals-14-02243-s001.zip › animals-3089821-supplementary.pdf]

Supplementary table S1. Feed composition and nutrient levels of the diet*

Ingredients (%)	Starter<br>0-7 days	Grower<br>8-15 days
Corn	38.97	45.87
Wheat	15	15
Soybean meal (42.6% CP)	32	25.6
Corn gluten	3	2.64
Meat and bone meal	2	2
Animal fat	4	3.88
Salt	0.25	0.25
Tricalcium phosphate	1.3	1.04
Limestone	1.26	1.22
Sodium bicarbonate	0	0.02
L-Threonine	0.12	0.16
Lysine	1.23	1.44
D-L-Methionine	0.33	0.03
Choline chloride (50%)	0.03	0.03
Premix#	0.2	0.2
Phytase	0.05	0.05
Feed additive	0.25	0.25
Anti-coccidia	0.01	0.01
Calculated nutrients		
Crude protein (%)	23	20.5
Crude fat (%)	6.31	6.36
Crude fibre (%)	3.01	2.8
Crude ash (%)	5.99	5.34
Calcium (%)	1.01	0.9
Available phosphorous (%)	0.6	0.53

Digestible lysine (%)	1.43	1.24
Digestible methionine + cystine (%)	1.07	0.95
Copper (ppm)	82.21	81.04
Zinc (ppm)	100.27	96.63
Metabolizable energy (kcal/kg)	3050	3150

* Feed was procured from Nonghyup Feed (Seoul, Korea).

[#] Trace minerals and vitamins provided in premix: Vitamin A, 12,000,000 IU; Vitamin D_3, 3,000,000 IU; Vitamin E, 40,000 IU; Vitamin K_3, 2,000 IU; Vitamin B_1, 2,000 mg; Vitamin B_2, 5,000 mg; Vitamin B_6, 3,000 mg; Vitamin B_{12}, 20 mg; Niacin, 40,000 mg; Pantothenic acid, 10,000 mg; Folic acid, 1,000 mg; Iron, 88,000 mg; Copper, 72,600 mg; Zinc, 60,000 mg; Manganese, 66,000 mg; Iodine, 990 mg; Selenium, 220 mg; Cobalt, 330 mg
